# Diseases of the Heart and Circulation

**Published:** 1901-10-05

**Authors:** 


### Diseases of the Heart and Circulation.

**Acute Dilatation of the Heart** in diphtheria, influenza, and rheumatic fever has recently been discussed in a valuable paper by Lees.[1] It is the cause of many if not most of the cases of sudden death occurring during and after diphtheria. The dilatation is due to acute fatty degeneration of the cardiac muscle, probably brought about by the direct action of the diphtheritic albumoses. It is of great importance to diagnose such a condition of cardiac dilatation, as fatal syncope may often be thus avoided. The clinical indications which should be sought for are—feebleness of the pulse wave, feebleness and diffusion of the cardiac impulse, extension of the cardiac dulness to the left, feebleness of the first sound at the apex with accentuation of the pulmonary second sound. These four indications of a weakened left ventricle are all present, more or less, in many cases of diphtheria. A fifth sign which is usually present also is a marked accentuation of the aortic second sound. This is often very decided, though the radial pulse is not tense, and one can only imagine that the tension in the aorta is raised by a contraction of the splanchnic arterioles through some central vasomotor irritation caused by the toxins. In a child suffering from diphtheria, as long as the cardiac dulness does not exceed one fingerbreadth outside the left nipple there is usually no immediate danger. If it exceeds two fingerbreadths there is urgent peril, and the child must not be allowed to sit up in bed for any reason whatsoever. The increase of dulness may sometimes be very rapid, and is frequently accompanied by vomiting, which is an important danger signal. The cardiac dilatation of diphtheria may occur at an early period of the illness, even after only a few days; or so late as six weeks from the commencement of the sore throat. The dilatation is probably seldom permanent, the less seriously damaged hearts probably again become of normal size, whilst the worst cases die. In influenza rapid dilatation of the heart frequently occurs, to a greater or less extent within a day or two after the onset of the disease, and it sometimes causes fatal syncope.

If the heart be examined there will often be found the above-mentioned signs of dilatation. As in diphtheria if the increase in dulness does not exceed one fingerbreadth outside the left nipple there is probably little danger, if it be two there is real danger. Minor degrees of cardiac dilatation after influenza may cause merely a feeling of incapacity for exertion, and without a careful examination of the heart such a patient may be considered a hypochondriac, and very injudicious advice be given. It is also certain that the dilatation caused by influenza may remain as a permanent dilatation, and give rise to very serious symptoms. In rheumatic fever, even in the most subacute attacks, acute dilatation of the heart seems to be in-invariably present; when the attack is over the dilatation lessens, and the cardiac dulness may again become of normal extent. Such dilatation, however, is far less dangerous than in either diphtheria or influenza. Thus an extension of the cardiac dulness to two fingerbreadths outside the left nipple implies in itself no immediate danger of death whatever. This is probably due to the fact that in diphtheria and influenza the muscular fibres of the left ventricle suffer greater destruction, while in rheumatism the myocardial changes are less intense, and one can only suppose that the elasticity of the ventricle is more affected. An extension of dulness more than two fingerbreadths beyond the left nipple may cause decided symptoms of collapse, and as in diphtheria a sudden vomiting is often the danger signal. An implication of the mitral valve allowing regurgitation into, and therefore increased tension within, the auricle has doubtless some share in the great enlargement of the left ventricle which may occur in the later stages of rheumatism in a child; but the damaged condition of the cardiac muscle is certainly the chief cause, and such cases should not be looked upon simply as "mitral regurgitation." Thus, in these three diseases, diphtheria, influenza, and rheumatic fever, acute dilatation of the heart may occur and may be the cause of sudden death. By careful palpation and percussion, however, the condition may be recognised, and such measures can be adopted as will, in very many cases, bring about recovery.

**The Diseases of the Heart in Middle and Advanced Life** have been discussed by Bruce[2] in the recent Lettsonian lectures. During the years between 20 and 45 the blood-pressure is relatively high, and the aorta and other large arteries increase in diameter from the stress of the blood-pressure on their elastic walls, and the heart increases in size year by year at a nearly uniform rate. After the age of 45, whilst the arteries continue to increase in circumference (somewhat more slowly than before) the blood-pressure falls and the heart begins to diminish in size—and these three features characterise the circulation for the next 20 years. This fall in the size of the heart is partly due to the widening of the arterial trunks, partly to lessening of mechanical stress due to comparative bodily relaxation and loss of vaso-motor tone in the splanchnic area. After the age of 65 the heart once more increases in size, owing to increase in the peripheral resistance brought about by a considerable proportion of the capillary network becoming obsolete; so that the heart at 75 is as large as it was at 45. The circulatory organs pass through these regular and normal changes, whilst injurious influences bring about pathological variations and actual disease. One of the chief of these is physical stress, either sudden violent exertion or ordinary laborious occupation. Many of these cases also present a history of strain during youth or early manhood, or a history of gout. Gout is one of the commonest causes of arterial degeneration, which lessens the elasticity of their walls. Nervous strain in the form of distress, worry, and axiety originate many cases of cardiac and arterial degeneration. It is again often found that cardiac disease in the latter half of life is due to extrinsic poisons—alcohol, tea, coffee, and lead. Syphilis, again, is a standing danger to the heart and arteries of the middle-aged man, and even in declining years. Emphysema and other chronic diseases of the lung and pleura strain the right ventricle, and chronic Bright's disease similarly strains the left ventricle. The last of the causes of disease of the heart and arteries in middle and advanced life is a premature senility, which often runs in families. The vital energy of such persons is exhausted prematurely; they are already old at 45, degeneration of their muscle and other cells setting in early.

[1] Brit. Med. Jour., Jan. 5, 1901. [2] Lancet, March and April 1901.

